# A robotic pill for oral delivery of biotherapeutics: safety, tolerability, and performance in healthy subjects

**DOI:** 10.1007/s13346-021-00938-1

**Published:** 2021-02-19

**Authors:** Arvinder K. Dhalla, Ziad Al-Shamsie, Simret Beraki, Anvesh Dasari, Leonard C. Fung, Laura Fusaro, Anusha Garapaty, Betsy Gutierrez, Delia Gratta, Mir Hashim, Kyle Horlen, Padma Karamchedu, Radhika Korupolu, Eric Liang, Chang Ong, Zachary Owyang, Vasudha Salgotra, Shilpy Sharma, Baber Syed, Mansoor Syed, April T. Vo, Radia Abdul-Wahab, Asad Wasi, Alyson Yamaguchi, Shane Yen, Mir Imran

**Affiliations:** Rani Therapeutics, LLC, 2051 Ringwood Ave, San Jose, CA 95131 USA

**Keywords:** Octreotide, Oral biotherapeutics, Oral biologics delivery, Robotic drug delivery platform

## Abstract

Biotherapeutics are highly efficacious, but the pain and inconvenience of chronic injections lead to poor patient compliance and compromise effective disease management. Despite innumerable attempts, oral delivery of biotherapeutics remains unsuccessful due to their degradation in the gastrointestinal (GI) environment and poor intestinal absorption. We have developed an orally ingestible robotic pill (RP) for drug delivery, which protects the biotherapeutic drug payload from digestion in the GI tract and auto-injects it into the wall of the small intestine as a safe, pain-free injection since the intestines are insensate to sharp stimuli. The payload is delivered upon inflation of a balloon folded within the RP, which deflates immediately after drug delivery. Here we present results from two clinical studies demonstrating the safety, tolerability and performance of the RP in healthy humans. In the first study, three versions of the RP (A, B and C) were evaluated, which were identical in all respects except for the diameter of the balloon. The RP successfully delivered a biotherapeutic (octreotide) in 3 out of 12 subjects in group A, 10 out of 20 subjects in group B and 16 out of 20 subjects in group C, with a mean bioavailability of 65 ± 9% (based on successful drug deliveries in groups A and B). Thus,  reliability of drug delivery with the RP ranged from 25 to 80%, with success rate directly related to balloon size. In a separate study, the deployment of the RP was unaffected by fed or fasting conditions suggesting that the RP may be taken with or without food. These promising clinical data suggest that biotherapeutics currently administered parenterally may be safely and reliably delivered via this versatile, orally ingestible drug delivery platform.

**Graphical abstract:**

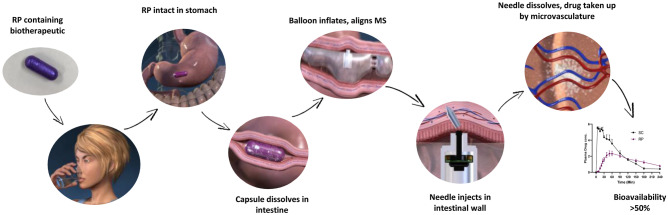

## Introduction

The use of biotherapeutics has been increasing exponentially since 1982 when the US Food and Drug Administration approved insulin, the first therapeutic recombinant protein [1]. Currently, there are more than 350 biologics commercially available for the treatment of various diseases. Because biotherapeutics are susceptible to digestion, they cannot be taken orally and must be injected parenterally [2]. Chronic parenteral administration of biotherapeutics represents a burden to patients because it interferes with their quality of life and compliance with therapy [3–7] and may be complicated by adverse events due to SC, IM, or IV injections [8–11]. Therefore, the convenience of oral delivery of biotherapeutics is a highly desirable goal for patients and caregivers as it can increase compliance and therapeutic outcomes [10, 12]. Despite numerous attempts, oral delivery of biologics has remained challenging because of the rapid breakdown and digestion of proteins by the gastrointestinal (GI) proteases and enzymes, and poor intestinal absorption [13–19]. Attempts at orally delivering small peptides using protease inhibitors and permeation enhancers have resulted in bioavailability of less than 1% [20–23].

We have designed a versatile, orally ingestible robotic pill (RP) for drug delivery which can deliver a number of biotherapeutics for multiple indications. The RP is a mechanical device, a robotic auto-injector enclosed in a standard pharmaceutical (methylcellulose) capsule shell, enteric-coated to prevent its dissolution and deployment in the acidic environment of the stomach. A precise dose of the sterile biotherapeutic (payload) is packaged inside a hollow, dissolvable needle loaded within a microsyringe, which itself is attached to a folded, self-inflating balloon. Once the RP reaches the small intestine, the pH change dissolves the enteric coating and the capsule shell, exposing the RP to intestinal fluid. This triggers a chemical reaction which leads to rapid inflation of the balloon, which aligns the microsyringe perpendicular to the long axis of the small intestine, and injects the dissolvable needle carrying the drug payload into the intestinal wall [24]. As the intestine is insensate to sharp nociceptive stimuli, the injection in the intestinal wall is expected to be painless [25, 26]. Previously, we have shown that the RP can reliably deliver biotherapeutics with high bioavailability in porcine and canine models [24, 27, 28]. This report describes the results of two clinical studies in healthy human subjects. The first study focused on evaluating the safety, tolerability, and performance of the RP in fasting subjects while tracking the bioavailability of octreotide (an approved therapeutic peptide for the treatment of acromegaly and neuroendocrine tumors) as an example of an injectable biotherapeutic drug. The second study assessed the safety, tolerability, and effect of food, if any, on the deployment of the RP platform alone, without a drug.

## Materials and methods

### RP description and operation

**Fig. 1:**
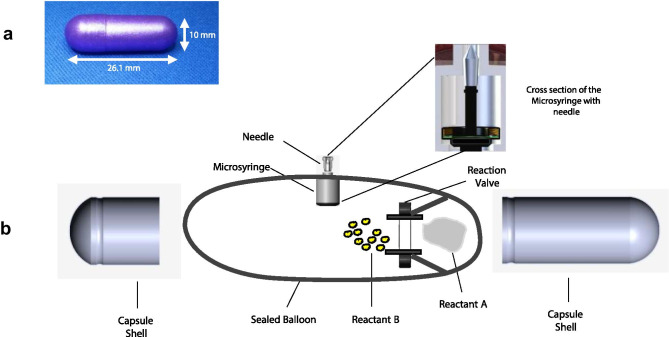
RP design. **a** Fully assembled enteric-coated RP. **b** Schematic drawing showing various parts and components of the RP. Inset shows the microsyringe containing the needle with the drug microtablet which gets injected into the jejunal wall. The microtablet and needle are aseptically manufactured in an isolator and hermetically sealed inside a drug chamber which is then inserted in the microsyringe

The RP (Fig. 1a) is a swallowable mechanical device enclosed in a 000-sized hydroxypropyl methylcellulose (HPMC) capsule (ACG Worldwide). Figure 1b is an expanded view showing major components of the RP which are enclosed inside a custom-designed polyethylene balloon with a length of 75 ± 2 mm and a diameter ranging between 21 and 25 mm. A cylindrical microsyringe (14.5 × 8.5 mm) made of polyethylene (inset, Fig. 1b) attached to the balloon contains the drug payload in solid form, sealed inside a dissolvable, hollow needle (5.1 ± 0.13 mm long) made of polyethylene glycol. Two reactants (citric acid and potassium bicarbonate) are kept separated inside the balloon by a dissolvable reaction valve made of polyethylene oxide which readily dissolves upon exposure to intestinal fluid. The capsule is enteric-coated using a Caleva mini coater (agitation 8–15 Hz, pump 1.3–15 rpm, atomizing pressure 10–20 psi) with a pH-sensitive polymer suspension of Eudragit L30-D55 (MW 320,000 g/mol) and 0.1–0.5% Plasacryl-HTP20 (Evonik).

The RP is ingested with water and stays in the stomach for a variable duration. The enteric coating protects the RP from dissolution in the acidic gastric environment (pH < 5.5). As the capsule enters the duodenum, the enteric coating and the HPMC capsule shell begin to dissolve at the higher intestinal pH (> 6), exposing the dissolvable reaction valve to the intestinal fluid. This leads to the reaction valve triggering the chemical reaction between citric acid and potassium bicarbonate to produce carbon dioxide (CO_2_). As the gas inflates the balloon and aligns the microsyringe perpendicular to the long axis of the intestine, pressure builds up and provides the force needed for the microsyringe to inject the needle into the intestinal wall. In the moist tissue environment, the needle and the drug dissolve within 10–15 min and the drug is rapidly absorbed into the bloodstream. Immediately upon needle delivery, the balloon deflates and is excreted through the GI tract with normal bowel movements.

The RP contains two radiopaque markers to track its transit, location, and deployment status in the GI tract using radiographic imaging. One of the two markers is barium sulfate powder which is compacted on one end of the capsule shell. Once the capsule shell dissolves, barium sulfate starts to disperse which indicates fluid ingress inside the capsule and imminent drug delivery (taken as T = 0 ± 5 min). Note that dispersion of barium sulfate only indicates that the RP has deployed (T = 0) but does not confirm if the drug was successfully delivered; confirmation of drug delivery (success or failure) is determined by subsequently analyzing the serial blood samples for presence or absence of drug. The second marker is in the form of a ring made of bismuth, placed at the base of the microsyringe which stays within the balloon and can be used to confirm the excretion of the device remnants.

Materials used in the device are classified as food grade, food additive, active, or inactive food ingredient or GRAS (generally recognized as safe) by the FDA.

### Test articles

RPs were manufactured by Rani Therapeutics, San Jose, CA, and were shipped to the study site at 2–8°C. In the first study (Study 1), three variations (A, B, and C) of the RP were evaluated in groups A, B, and C. The three configurations of the RP differed in the size of the balloon, with RP A containing the smallest of the three with a diameter of 21 mm, RP B with a diameter of 23 mm, and RP C with a diameter of 25 mm. The rest of the components and mechanism of operation of the devices were the same in all three versions. The dose of octreotide was 100 µg in all 3 versions. Octreotide acetate was procured from Bachem (Switzerland) and lyophilized under GMP conditions at the University of Iowa Pharmaceuticals (Iowa). The lyophilized powder was compacted into microtablets containing 100 µg of octreotide, inserted and sealed inside the dissolvable needles, and enclosed into the needle chamber under aseptic conditions within an isolator. The needle and drug were certified to be sterile by sterility testing done by an external CRO (Pacific Bio Labs, Hercules, CA).

For the IV group, the octreotide (Sandostatin®) formulation (Lot# PPP.19.750 manufactured by Novartis Pharma Stein AG) used in the study was purchased commercially in 1 mL ampules at a concentration of 100 µg of octreotide per 1 mL.

**Fig. 2:**
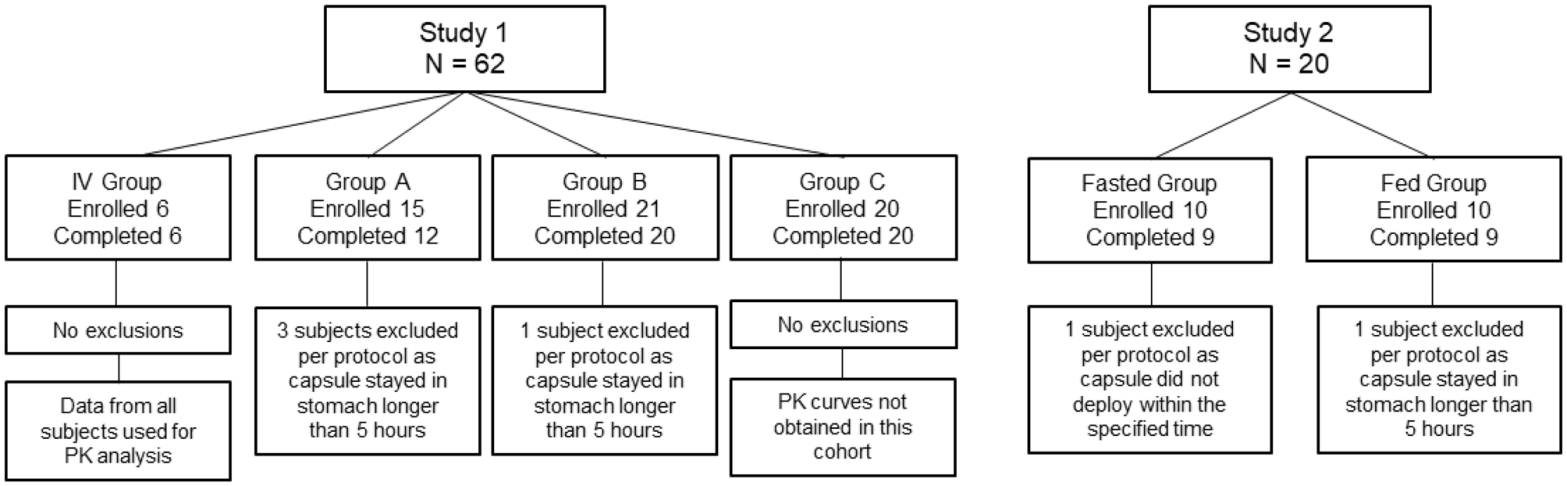
Overview of the study design and assignment of participants to different groups

In the second study (Study 2), the RPs were of the same configuration as version A in Study 1, but did not contain any drug or needle. Figure 2 shows the overall design and subject assignments of the two studies.

### Study 1 objectives and endpoints

The primary objectives of this study were to evaluate the safety, tolerability and reliability of the RP in healthy human subjects using octreotide as an example for the delivery of a biotherapeutic. Safety and tolerability metrics included documenting any pain or discomfort experienced by the subjects during the transit of the RP in the GI tract or upon deployment. This was recorded by each subject completing a questionnaire about their experience after taking the RP. Fluoroscopic imaging at the end of the study was done to confirm that RP remnants were safely expelled. Reliability metrics included drug delivery success rate which was determined by the presence or absence of the peptide, octreotide, in serial plasma samples taken from each subject during the study period.

The secondary endpoint of the study was to determine the absolute bioavailability (% F) of octreotide delivered via the RP. This was done in groups A and B by frequent sampling following T = 0 in order to get complete pharmacokinetic (PK) profiles of octreotide.

### Study 1 design

This was a single-center, open-label study. Eligible, consenting subjects were assigned to receive 100 µg of octreotide delivered via the orally ingested RP (*N* = 56), wherein the drug was formulated as a solid tablet and injected into the jejunal wall or IV injection (*N* = 6). Three variations of the RP (with increasing balloon sizes) were tested in the three subgroups as follows: group A (*N* = 15), group B (*N* = 21), and group C (*N* = 20).

### Study 1 population

Healthy male or female subjects aged 18–55 years with a BMI of 19–32 kg/m^2^ were eligible for inclusion in the study. Key exclusion criteria for all subjects included past or active GI disorders including diarrhea, constipation or other manifestations suggestive of abnormal GI function. Regular use of antacid, proton pump inhibitor (e.g., omeprazole) or histamine H_2_ receptor antagonists or any single use within 5 days of the study day was prohibited. The study protocol was reviewed and approved by the relevant Human Research Ethics Committee and the study was conducted in accordance with Good Clinical Practice per National Statement on Ethical Conduct in Human Research produced by the National Health and Medical Research Council of Australia. Written, informed consent was obtained from all subjects to participate in the study.

### Study 1 procedures

#### RP groups

Subjects (*N* = 56) assigned to the 3 RP groups were admitted in the fasting state and, immediately after the collection of a baseline peripheral blood sample, were instructed to swallow a single RP containing 100 µg of octreotide with water. The objectives of groups A and B were (1) to determine the success rates (reliability or performance) corresponding to the 2 different sized balloons in groups A and B and (2) to obtain full PK in profiles to determine the absolute bioavailability (%F) of octreotide delivered via the RP. Accordingly, in groups A and B, the transit of the RP was tracked fluoroscopically at frequent intervals to ascertain the gastric emptying time (GET) and intestinal deployment time (IDT). Fluoroscopic imaging was performed with Siemens AXIOM Artis dMP using standard abdominal protocol. The GET was measured as the time from RP ingestion to the time the RP was confirmed to be in the small intestine, and the IDT was determined by measuring the time the RP was confirmed to be in the small intestine to its time of deployment. Upon confirmation of device deployment (T = 0), serial blood sampling was initiated for determination of plasma concentrations of octreotide at 5, 10, 15, 20, 25, 30, 40, 50, 60, 90, 120, 150, 180, and 240 min.

In the case of group C, which had the largest sized balloon, the objective was solely to determine the reliability (the success rate of drug delivery). Accordingly, the blood samples were collected hourly between 2 and 10 h after the ingestion of the RP. The drug delivery via RP was considered successful when the presence of octreotide was confirmed in one or more of the hourly samples. This protocol helped to greatly reduce the subjects’ exposure to frequent X-rays, as monitoring was limited to a single image taken 7–8 h after the RP ingestion to verify that the device had deployed.

In all 3 groups, fluoroscopic imaging was performed on the next day after dosing, any adverse effects recorded, and the study participants were discharged. Prior to discharge, subjects were instructed to complete a questionnaire about their experience with the ingestion of the device and their perception of any pain or discomfort. Subjects returned for a follow up medical examination on day 3 and day 7 to fluoroscopically confirm excretion of RP remnants.

#### IV group

Subjects (*N* = 6) were admitted in the fasting state and received a single 100 µg IV injection of octreotide (Sandostatin®). Serial blood samples for PK analysis were collected immediately before the injection and at 5, 10, 15, 20, 25, 30, 40, 50, 60, 90, 120, 150, 180, and 240 min after dosing.

#### Octreotide plasma bioanalysis

Blood samples were collected into potassium-EDTA treated lavender top tubes, plasma was separated via centrifugation and aliquots stored at −80℃ until analyzed. Plasma octreotide concentrations were analyzed by PHARMout Labs (Fremont, CA) using an LC/MS/MS method, validated according to applicable current guidelines for octreotide quantification in human plasma over the concentration range of 0.2 to 50 ng/ml. The CV% obtained for inter and intra assay was 5.4% to 7.1% and 1.3% to 13.6%, respectively. The analysis was performed using an API 5000 mass spectrometer from Applied Biosystems.

#### Study 2 objectives and endpoints

This study was conducted with the RP device without a drug or needle in healthy human volunteers to evaluate the effect of food, if any, on the transit and deployment of the RP in the GI tract. Accordingly, both in the fed and fasted states, subjects’ experience with the RP was evaluated along with a number of metrics including the robustness of the enteric coating to withstand the acidic gastric environment in the fed *vs.* fasted state, and any changes in the transit and deployment times of the device, as measured by the GET and IDT.

#### Study 2 design

This was a single center, open-label, non-significant risk study. Subjects were randomly assigned to either a fasting group (*N* = 10) or a postprandial group (*N* = 10). A single RP (without a needle or drug) was administered to each subject. The fasting group swallowed the RP with 6 oz. of water after a ≥ 10-h fast. Subjects in the postprandial group were given a standardized meal after an overnight fast of ≥ 10-h, and 45 min later swallowed the RP with 4 oz. of water.

#### Study 2 population

Healthy male or female subjects (*N* = 20) aged 20–50 years with a BMI < 28 kg/m^2^ were eligible for inclusion in the study. Key exclusion criteria for all subjects were past or active GI issues including but not limited to chronic diarrhea, chronic constipation, gastroparesis/delayed gastric emptying, gastric or duodenal ulcer, dysphagia, dyspepsia, esophagitis, esophageal spasm, bulimia nervosa, gastroesophageal reflux disease, irritable or inflammatory bowel disease, Crohn’s disease, diverticulitis, ischemic, or ulcerative colitis. Regular use of antacid, proton pump inhibitor (e.g., omeprazole) or histamine H_2_ receptor antagonists (e.g., ranitidine) or any single use within 5 days of the study day was prohibited. All subjects granted their written, informed consent to participate in the study. The study was conducted in accordance with Good Clinical Practice under a protocol approved by an IRB.

#### Study 2 procedures

 The transit of the RP through the GI tract, after its ingestion, was tracked by serial abdominal radiographic imaging in all subjects to determine the GET and IDT, in the same manner as described for Study 1. In the Fasted group, radiographic imaging of the abdominal cavity was done at 20-min intervals while the RP was in the stomach. Once the RP entered the small intestine, the imaging intervals were shortened to 15 min until confirmation of device deployment was visualized by the dispersion of the barium sulfate.

In the Fed group, subjects were given a standardized meal, including an egg sandwich (consisting of 2 eggs and 2 pieces of toast/bread) and 4 oz. of orange juice, after fasting for ≥ 10 h before their arrival at the study site. The RP was administered with 4 oz. of water 45 min after the meal was provided. Upon confirmation of RP ingestion, radiographic images of the abdominal cavity were acquired every 30 min until RP deployment in the small intestine. Imaging was discontinued per protocol if the RP remained in the stomach for > 5 h.

After confirmation of the RP deployment, subjects from all groups were instructed to complete a questionnaire probing their experience with the ingestion of the device and their perception of any pain or discomfort, and their vital signs were recorded. The subjects returned 3 or 4 days after the study day for a final abdominal radiographic image to confirm that the RP had been excreted.

#### Data analysis

MS Excel (2016) and GraphPad (Version 8.4.3) were used for data analysis. Categorical data are expressed as counts and percentages, and continuous data as mean ± standard deviation (SD) or standard error (SE), as indicated. PK parameters were calculated using noncompartmental analysis (NCA) implemented within a validated installation of Phoenix® WinNonlin. The C_max_ (peak plasma concentration) and T_max_ (time to reach C_max_) were obtained graphically from the concentration *vs*. time profiles. Unpaired *t* tests were used to determine differences between groups with a *p* value of 0.05 or less deemed as significant.

## Results

### Study 1

#### Subject characteristics

A total of 62 healthy subjects (35 men, 27 women) were enrolled and randomly assigned to one of the four study groups designed to receive a single dose of octreotide either orally through one of the three versions of the RP (group A: *N* = 15; group B: *N* = 21; group C: *N* = 20) or via IV injection (*N* = 6). Enrollment and group assignment details are shown in Fig. 2, with demographics of the subjects shown in Table 1. All subjects were healthy and free from past or active GI dysfunction. The age and body mass index (BMI) of subjects were similar within each group, and three of the four study groups enrolled a majority of male volunteers (Table 1). Of the 62 subjects enrolled, 58 subjects (94%) completed the study; 4 subjects were removed from the study due to GET being greater than 5 h according to the protocol.

**Table 1:** Demographics of the study groups

	Study 1				Study 2	
Characteristic	IV Group (N = 6)	RP A (N = 15)	RP B (N = 21)	RP C (N = 20)	Fasted Group (N = 10)	Fed Group (N = 10)
Age (years)						
Mean (SD)	26 (7)	26 (5)	27 (5)	27 (9)	26 (4)	36 (11)
Range	18–35	18–33	19–35	18–51	20–33	20–49
Gender, N (%)						
Male	4 (67)	10 (67)	13 (62)	8 (40)	6 (60)	5 (50)
Female	2 (33)	5 (33)	8 (38)	12 (60)	4 (40)	5 (50)
BMI (kg/m^2^)						
Mean (SD)	22.8 (2.7)	24.9 (3.2)	24.0 (3.0)	22.9 (2.9)	24.9 (2.3)	24.3 (2.3)
Range	9.1–26.5	19.6–30.4	19.9–30.4	19.0–27.8	21.6–27.3	20.1–27.9

*SD* standard deviation, *BMI* body mass index

#### RP deployment times

**Fig. 3:**
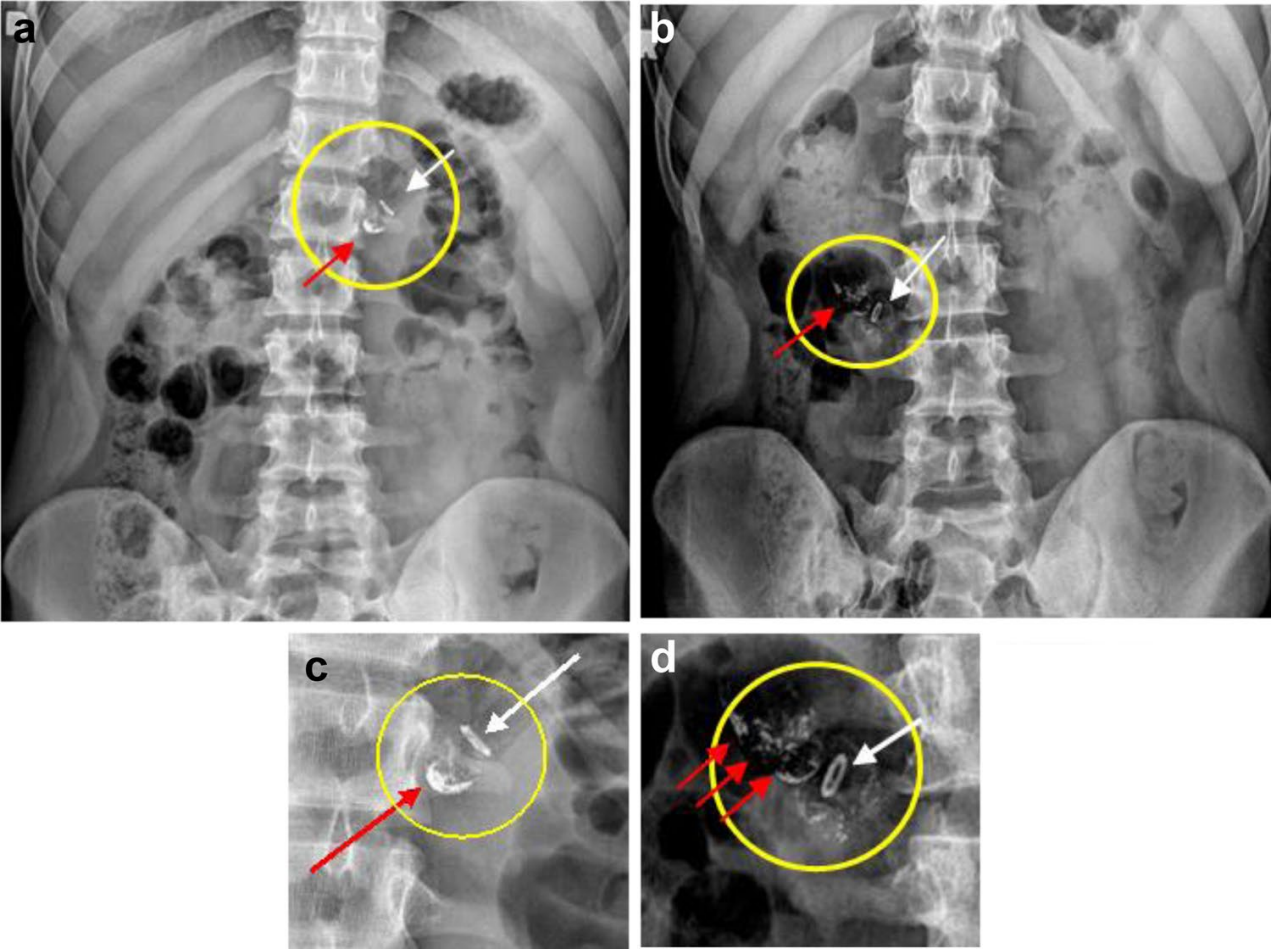
**a** Representative X-ray image of an intact RP residing in the stomach (encircled) showing a radio-opaque ring (which is part of the device) at one end of the device (white arrow) and barium sulfate powder inside the capsule shell at the other end (red arrow). **b** Representative X-ray image of a deployed RP in the small intestine (encircled). The radio-opaque ring (white arrow) is part of the device whereas barium sulfate is dispersed inside the intestinal lumen (red arrows). **c **Magnified encircled area from **a**. **d** Magnified encircled area from **b**.

Passage of the RP was tracked via frequent fluoroscopic imaging in all subjects in groups A and B. Sample images are shown depicting an intact RP in the stomach (Fig. 3a), and a deployed RP in the proximal segment of the small intestine (Fig. 3b). The RP deployment time was determined in 32 of 36 subjects in groups A and B (*N* = 12 of 15 from group A, *N* = 20 of 21 from group B). Four subjects in which the RP remained in the stomach for more than the time allowed per protocol were subsequently removed from the study. The mean (± SE) time for RP deployment from the time of ingestion was 205 ± 16 min in group A (*N* = 12) and 260 ± 18 min in group B (*N* = 20). The average (± SE) GET was 114 ± 18 and 142 ± 16 min for groups A and B, respectively. The average (± SE) IDT was 91 ± 6 and 118 ± 10 min for groups A and B, respectively. GET and IDT were not determined in Group C per protocol.

#### Device safety and tolerability assessment

The RP was well-tolerated by all subjects. None of the subjects had any difficulty or issues swallowing the RP. There were no serious adverse effects reported by any of the subjects. A total of 29 adverse events of grade levels 1 and 2 (mild to moderate) were reported by 23 subjects, all of which resolved shortly after onset without any intervention. The majority of the adverse events observed (lightheadedness, diarrhea, headache, and nausea) were likely a result of the prolonged fasting duration and/or known side-effects of octreotide.

One subject in group B and two subjects in group C noted abdominal discomfort, which resolved on its own and no intervention was required. After further analysis of each case, it was found that the onset of abdominal pain did not coincide with the timing of device deployment and was thus deemed unrelated to the RP. Fluoroscopic imaging done between days 3–7 at the follow-up visits confirmed the excretion of device remnants from the GI tract without sequelae in all subjects. All physical examinations and clinical laboratory tests remained normal throughout the duration of the study.

#### Device reliability (drug delivery success rate)

**Fig. 4:**
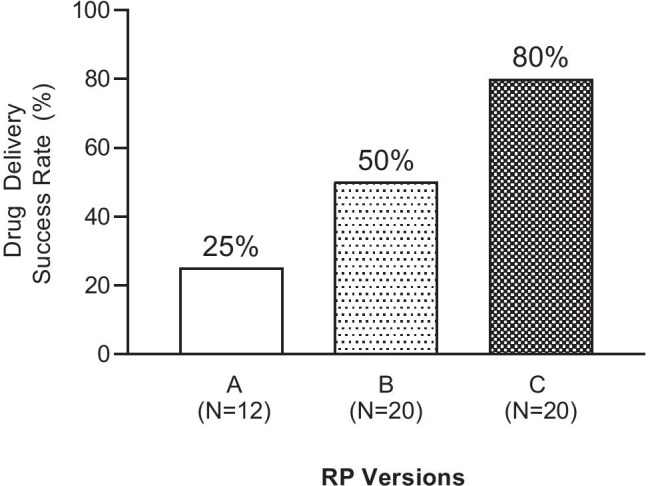
Drug delivery success rate with different versions of the RP. Successful delivery was defined as presence of detectable levels of octreotide in at least one plasma sample. Numbers in parentheses indicate total number of subjects in that group

Reliability of the RP was scored on the basis of successful or unsuccessful drug delivery, which was determined by the presence or absence of octreotide in at least one of the blood samples collected from each subject in the three RP groups. Figure 4 shows the success rate for drug delivery of the three RP versions. There was a progressive increase in success rate with the three versions of the RP; with the lowest rate (3 out of 12 successful deliveries or 25%) observed in the smallest size RP (group A), a higher rate (10 out of 20 successful deliveries or 50%) in the intermediate size RP (group B) and the highest rate of 80% (16 out of 20 successful deliveries) in group C with the largest balloon size.

#### Pharmacokinetics and bioavailability of octreotide delivered via the RP

**Fig. 5:**
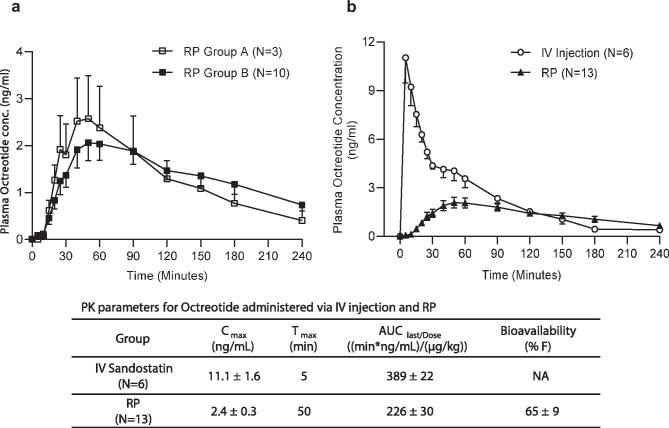
PK of octreotide in healthy human volunteers. **a** Time-course of changes in plasma concentrations of octreotide delivered via RP A and B. **b** Time-course of changes in plasma octreotide levels following octreotide administration either IV (*N* = 6) or orally via the RP (*N* = 13, groups A and B combined) in healthy human volunteers. Numbers in the table below the graphs are PK parameters for the IV and RP groups. Data are presented as means ± SE

As indicated in the preceding section on reliability, complete PK curves were obtained in 3 subjects in group A and 10 subjects in group B (Fig. 5a). Since the drug dose was identical across groups A and B, data were pooled (*N* = 13) for PK analyses and determination of bioavailability of octreotide. The concentration–time profiles of octreotide administered via IV injection and RP are shown in Fig. 5b. Peak plasma concentration (C_max_) of octreotide was higher with IV administration (11.1 ng/ml) compared to RP (2.4 ng/ml). The time to reach peak plasma concentration (T_max_) was shorter for IV (5 min) compared to RP (50 min) as expected. Exposures were higher in subjects administered octreotide IV when compared to oral administration, with a mean AUC_last/dose_ value 1.7-fold higher in the IV group compared to the RP group (389 vs. 226 min ng/mL µg/kg). The mean bioavailability (% F) of octreotide delivered via the RP, calculated using weight-normalized AUC, was 65 ± 9%. Variability (geometric coefficient of variation (CV%)) across all PK parameters with the RP was moderate (range 21 to 92%) and similar to that observed in the IV group (range 13 to 72%).

### Study 2

#### Subject characteristics

A total of 20 subjects participated in this study and each received a single RP, either in a fasting state (*N* = 10, the “Fasted Group”) or postprandial state (*N* = 10, the “Fed Group”). Demographics of the study participants are presented in Table 1. All subjects were healthy and free from past or active GI dysfunction.

#### Safety and tolerability evaluation

No subject reported any perception during the transit of the RP through the GI tract and/or during the deployment of the device. Abdominal imaging conducted during a return visit of the subjects (72–96 h after ingestion) confirmed that all device remnants had been excreted without sequelae in all subjects.

#### RP deployment times

Passage of the RP was tracked via frequent radiographic imaging in all subjects. GET was determined in 19 of the 20 study subjects. In one subject in the Fed Group, the RP remained intact in the stomach for 300 min after ingestion, and serial imaging was discontinued per protocol. IDT was measured in 18 of the 20 study subjects. Another RP in the Fasted Group remained intact inside the small intestine approximately 390 min (6.5 h) after its ingestion. Imaging of this second RP was discontinued per protocol.

**Fig. 6:**
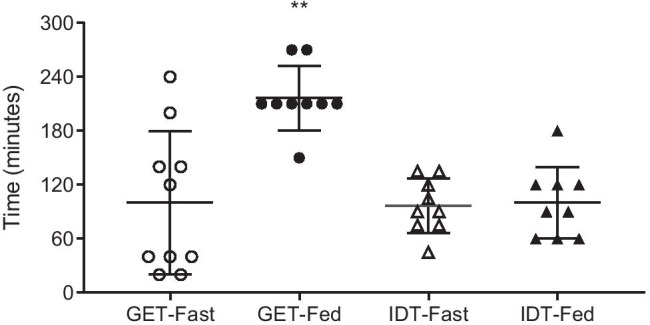
Gastric emptying time (GET) and intestinal deployment time (IDT) of the RP in fasted and fed healthy subjects. Each point corresponds to individual values for each subject. Horizontal lines are means ± SE for the respective groups. **p < 0.0009 compared to the fasted group

The average (± SE) time to RP deployment from its ingestion was 181 ± 29 min in the Fasted Group and 317 ± 17 min in the Fed Group. The mean GET was significantly (*p* < 0.05) shorter (100 ± 25 min) in the Fasted Group than in the Fed Group (217 ± 12 min) (Fig. 6). The average (± SE) IDT was 97 ± 10 min in the Fasted Group and 100 ± 13 min in the Fed Group, which were not statistically different (*p* = 0.84) (Fig. 6).

## Discussion

The results described herein are the first studies in humans demonstrating safety, tolerability, and effectiveness of an orally ingestible drug delivery device autonomously injecting a peptide into the human intestinal wall at clinically relevant doses with a bioavailability rivalling that of parenteral injections.

Octreotide, a representative candidate for drug delivery by the RP, is the first biotherapeutic that we tested from bench to humans. The results of this proof-of-concept study are important, as they represent the potential of this platform to deliver any payload that can be accommodated within the capacity of the needle which in its current configuration is about 3.5 mg.

Multiple approaches have been explored to enable oral delivery of proteins and peptides, from co-administration of substances which modify the permeability of the GI tract to delivery of drugs along with a carrier or with mechanical devices [13–17, 24, 29–32]. The most common approaches entail the use of protective enteric coatings, protease inhibitors, and intestinal permeation enhancers [21, 33]. These absorption enhancers work by damaging the mucosal lining of the intestine which can lead to dose variability and potential safety concerns with chronic use, as repeated damage to the intestinal epithelium could impair its protective barrier function and result in intestinal ulcers [34]. While the abovementioned strategies have shown success for oral formulation of some biologics, the bioavailability using these strategies remains low (≤ 1%), limiting the approach to a few small peptides [14, 20, 23].

In contrast, development of the ingestible RP represents a marked departure from such earlier attempts at promoting the oral delivery of large biopharmaceuticals. Instead of modifying the drug to facilitate its enteral absorption, the drug in the RP is protected throughout its transit until it reaches the small intestine, where it is robotically injected into a richly vascularized territory. Using this approach, we have previously demonstrated the successful delivery of a number of peptides and antibodies via the RP in experimental animal models [24, 27, 28].

Similar attempts have been made to create devices that would auto-inject drugs into the GI tract, however, a reliable and consistent approach has not been demonstrated. A capsule-like device with hollow needles protruding out, endoscopically deposited into the small intestine of anesthetized swine, was shown to deliver the drug as the needles passively penetrate the intestinal wall during peristaltic contractions [29]. No further work has been reported on this device. More recently, the same group reported data on another device called SOMA, which could inject small doses of insulin via spring-loaded needles, when endoscopically deposited in the stomach of fasted, anesthetized pigs [32]. Both of these early experimental approaches failed to demonstrate consistent drug delivery success despite endoscopically depositing these devices in the GI lumen under highly controlled testing conditions. In addition, the authors have postulated, but not demonstrated, a viable approach to delivering these devices to discrete locations within the GI tract (stomach or jejunum).

### Safety and tolerability of the RP

Data from both studies described herein demonstrated that the RP was easily swallowed, withstood the acidic gastric environment, deployed painlessly once in the alkaline milieu of the small intestine, and was properly excreted with normal bowel movements. The hollow organs of the GI tract are insensate to typical noxious stimuli, such as puncturing, cutting, pinching and burning, while notoriously sensitive to stretch and distension [26]. The absence of any reported pain or discomfort during deployment of the RP from any of the subjects suggests that the transient balloon inflation and deflation is insufficient to activate the intestinal stretch receptors.

### Reliability (drug delivery)

Three versions of the RP, with the balloon diameter increasing by about 2–4 mm between versions, were tested in Study 1. We started with the smallest balloon size in the first version (RP A) out of an abundance of caution to ensure that there was no perception of pain or discomfort due to the activation of intestinal stretch receptors when the balloon inflates and pressurizes transiently during intestinal deployment. Progressive increase in success rate with increasing balloon size confirmed that balloon size was a key determinant of success rate for drug delivery. Of the three versions of the RP, version C which had the largest sized balloon yielded the highest (80%) drug delivery success rate, correlating balloon size to success rate. Although the reasons for failures are not fully understood, we speculate that one of the causes for failure may be that capsule deployment occurred but the needle failed to penetrate the intestinal wall due to misalignment of the microsyringe to the intestinal wall. Other reasons for failures may be due to errors during the manufacturing processes, which are currently being done manually. We expect that the success rate will further improve with balloon size optimization and reduction of manufacturing defects and inconsistencies with fully automated manufacturing.

### Bioavailability of octreotide delivered via RP

PK analysis of successfully deployed RPs from groups A and B (*N *= 13) indicated that octreotide delivered by the RP yielded an absolute bioavailability of 65%, far surpassing the ≤ 1% achieved to date with previous attempts [20, 23]. The subjects which showed no drug levels were designated as failures of device performance (these data are captured in device reliability), thus were not included in the PK analysis. PK data from this clinical study with the RP are consistent with our previous preclinical data showing high bioavailability of several molecules on par with that observed via parenteral routes [27, 28]. For example, a preclinical study with the RP in awake dogs yielded 78% absolute bioavailability for octreotide [unpublished data]. The inter-subject variability in the PK profiles of octreotide delivered via the RP was similar to that observed in the IV group and consistent with previous reports [35].

It is noteworthy that both the high reliability (80%) as well as the high bioavailability (65%) demonstrated here for an orally delivered biotherapeutic are unprecedented. For context, the success rate with octreotide delivered via the RP is about twofold higher and bioavailability 65-fold greater than that of the recently approved oral version of the GLP-1-mimetic peptide, semaglutide (*Rybelsus®*), with a mean success rate of ~ 40% and a bioavailability of ~ 1% [23]; however, the low reliability and bioavailability of semaglutide are offset by a long half-life (7 days) and a wide therapeutic index such that the peptide met the clinical safety and efficacy endpoints with a daily dosing regimen. This example illustrates that, besides performance measures of reliability and bioavailability, drug properties such as half-life and therapeutic index are key considerations in the development of a safe and effective oral dosing regimen of a specific molecule and a specific indication.

### Effect of food on RP deployment

A key finding from Study 2 is that there was no effect of food on the device deployment time. While the mean GET was significantly prolonged in the fed state, there was no difference in the mean IDT between the Fasted and the Fed groups, indicating that the presence of food does not interfere with the dissolution of the enteric coating or the deployment time of the RP. This is important as some of the other oral delivery mechanisms are significantly affected by the presence of food. For example, subjects need to take oral semaglutide at least 30 min before the first food, beverage, or other oral medications of the day with no more than 4 oz of plain water. In addition, change in wait time before the medication can be taken could significantly affect the drug levels (*Rybelsus Prescribing Information*). Another recently approved oral peptide with less than 0.5% bioavailability, octreotide (*Mycapssa®*), is also recommended to be taken on an empty stomach or at least one hour before a meal or 2 h after a meal as the presence of food significantly affects drug absorption [36]. This is anticipated, as the delivery mechanism for both *Rybelsus* and *Mycapssa* rely on absorption enhancers to enhance bioavailability, which can be affected to a variable degree by the presence of food. Such restrictions and limitations on dosing can negatively impact patient compliance and/or can lead to variability in drug exposures impacting clinical outcomes. In contrast to these approaches, the RP offers an advantage because the presence of food does not affect its deployment. Further studies of drug delivery via RP in the presence of food are planned to confirm these findings.

### Limitations of the studies

One limitation of Study 1 is the small number of subjects with complete PK profiles. However, the PK profile of the drug is not expected to change with changes in device configuration as evidenced by no differences in the PK profiles of octreotide between groups A and B. Thus, data from the two groups was combined. A limitation of Study 2 is that while time to deployment (IDT) was not affected by food, actual drug delivery via the RP could not be determined. Data from our recent nonclinical studies confirm that PK of the drug delivered via RP is not different in fed vs. fasted animals. We are currently planning to conduct similar studies in humans.

### Limitations of the RP technology

From a clinical perspective, a limitation of the RP technology is the lack of predictability of the exact time of drug delivery following oral ingestion, due to the inherent variability in gastric residence times in human subjects [37, 38]. While this is not an issue for the majority of approved therapeutic applications, the unpredictability of the exact time of drug delivery may preclude the use of the RP for delivery of temporally sensitive biotherapeutics such as mealtime insulin.

Another limitation of the RP technology is the amount of drug payload it can accommodate, which is defined by the capacity of the single needle, which is currently 3.5 mg. Barring biologics which are administered frequently at high doses, the current capacity of the RP needle is sufficient to accommodate many of the approved injectable biotherapeutics with daily doses in the microgram or low milligram range, such as GLP-1 mimetics, basal insulin, octreotide, PTH, human growth hormone, factor VIII, and many enzyme-replacement therapies for rare diseases. Even injectables with a long half-life currently administered at high doses infrequently (weekly, biweekly or monthly) can be accommodated on the RP platform with a daily dosing regimen using much smaller doses. For example, adalimumab currently injected at a dose of 40 mg every 2 weeks can be converted to a daily dosing regimen with fractional daily dose of ~ 3 mg. The advantage with smaller, daily oral doses, besides convenience, is that drug exposures remain within a narrow therapeutic range, reducing the potential of adverse effects due to higher exposures.

## Conclusions

In conclusion, these initial human clinical studies demonstrate the safety and performance of a versatile, orally ingestible drug delivery platform in healthy human volunteers. The RP was safe, well-tolerated and delivered therapeutic amounts of octreotide (a biotherapeutic) with an unprecedented bioavailability of 65%, far exceeding the approximately 1% bioavailability with the current state-of-the-art therapy in oral biotherapeutic drug delivery. The safety and reliability of the RP technology remain to be determined in larger, long-term studies with repeat administrations in chronically ill patients and across other demographics. Nevertheless, it is noteworthy that in these single administration studies, there was no reported incidence of pain or discomfort associated with the RP either during its deployment or during needle delivery, and all device remnants were safely and uneventfully excreted in all subjects. If confirmed in larger patient populations after  repeat administrations with other biotherapeutic payloads, this innovative drug delivery platform may offer an oral alternative for many patients with chronic diseases currently taking frequent and painful parenteral injections.

## Data Availability

The datasets generated during and/or analyzed during the current study are available from the corresponding author on reasonable request.
